# Kidney-type glutaminase (GLS1) is a biomarker for pathologic diagnosis and prognosis of hepatocellular carcinoma

**DOI:** 10.18632/oncotarget.3196

**Published:** 2015-03-26

**Authors:** Decai Yu, Xianbiao Shi, Gang Meng, Jun Chen, Chen Yan, Yong Jiang, Jiwu Wei, Yitao Ding

**Affiliations:** ^1^ Department of Hepatobiliary Surgery, The Affiliated Drum Tower Hospital, Medical School of Nanjing University, Nanjing, 210093, China; ^2^ Jiangsu Key Laboratory of Molecular Medicine, Medical School of Nanjing University, Nanjing, 210093, China; ^3^ Department of Pathology, The Affiliated Drum Tower Hospital, Medical School of Nanjing University, Nanjing, 210093, China; ^4^ Nanjing University Hightech Institute at Suzhou, Suzhou, 215123, China; ^5^ Department of Hepatobiliary Surgery, The Third Affiliated Hospital of Soochow University, Changzhou, 213003, China

**Keywords:** kidney-type glutaminase (GLS1), liver-type glutaminase (GLS2), hepatocellular carcinoma, biomarker

## Abstract

The lack of sensitive and specific biomarkers hinders pathological diagnosis and prognosis for hepatocellular carcinoma (HCC). Since glutaminolysis plays a crucial role in carcinogenesis and progression, we sought to determine if the expression of kidney-type and liver-type glutaminases (GLS1 and GLS2) were informative for pathological diagnosis and prognosis of HCC. We compared the expression of GLS1 and GLS2 in a large set of clinical samples including HCC, normal liver, and other liver diseases. We found that GLS1 was highly expressed in HCC; whereas, expression of GLS2 was mainly confined to non-tumor hepatocytes. The sensitivity and specificity of GLS1 for HCC were 96.51% and 75.21%, respectively. A metabolic switch from GLS2 to GLS1 was observed in a series of tissues representing progressive pathologic states mimicking HCC oncogenic transformation, including normal liver, fibrotic liver, dysplasia nodule, and HCC. We found that high expression of GLS1 and low expression of GLS2 in HCC correlated with survival time of HCC patients. Expression of GLS1 and GLS2 were independent indexes for survival time; however, prognosis was predominantly determined by the level of GLS1 expression. These findings indicate that GLS1 expression is a sensitive and specific biomarker for pathological diagnosis and prognosis of HCC.

## INTRODUCTION

HCC occurs mainly in patients with chronic liver disease such as hepatitis B or C infection. It is challenging to distinguish early stage HCC from cirrhotic or dysplastic nodules in pathological biopsy material. Based on molecular profiling, several markers for early malignant HCC are also used in clinic. Glutamine synthetase (GS), glypican-3 (GPC3), and heat shock protein 70 (HSP70) have been validated, and can be used for histopathological diagnosis [1, 2]. However, highly sensitive and specific pathological or prognostic biomarkers for HCC diagnosis have not been developed, and their absence hinders pathological diagnosis and prognosis.

Metabolic deregulation has been considered a crucial hallmark of cancer [3, 4]. Increased aerobic glycolysis (also known as the Warburg effect) and glutaminolysis are commonly found in many malignancies [5, 6]. During malignancy development and progression, the glutamine (Gln) pathway provides a variety of essential products to sustain biological function and cell proliferation, such as ATP generation and macromolecules for biosynthesis [6–8].

Mitochondrial glutaminase is the key enzyme that converts glutamine to glutamate in glutaminolysis [9]. It plays a crucial role in regulating cellular catabolism and maintaining redox balance in cancer cells [10–12]. The *GLS1* gene, located in chromosome 2, encodes two isoforms, kidney-type glutaminase (KGA, long transcript isoform) and the glutaminase C (GAC, short transcript isoform), which are expressed in kidneys and in a variety of other tissues including cancer cells [13]. The *GLS2* gene, located in chromosome 12, encodes two isoforms, liver-type glutaminase (LGA, short transcript isoform) and glutaminase B (GAB, long transcript isoform), which are highly expressed in normal adult liver [13]. GLS1 is upregulated in cells with increased rates of proliferation, and accounts for the majority of glutaminase activity in some human tumor cells; whereas, GLS2 expression is associated with resting or quiescent cell states [14]. Previous studies have shown that GLS1 expression is upregulated in gliomas, colorectal carcinomas, adenomas, and breast cancer cell lines [15–17]. It has also been shown that GLS1 and GLS2 activities were increased approximately 20-fold and 6-fold, respectively, in transplanted hepatomas [18, 19]. Similar results were obtained in various hepatoma cells of human and rat origin in cultured form or in ascitic form [20]. Interestingly, glutamine metabolism can be induced by MYC to switch from GLS2 to GLS1 in mouse liver tumors [21]. These results highlight the importance of glutaminase in cancer, including HCC. Nonetheless, no specific associations between clinical outcomes and GLS1 or GLS2 expression have been identified.

We hypothesized that altered glutaminolysis could be used as a pathological biomarker for HCC. Therefore, we investigated the expression and distribution of GLS1 and GLS2 in a large set of HCC specimens. The results validated the relevance of GLS1 and GLS2 expression to HCC oncogenic transformation and clinical outcomes. We found that high expression of GLS1 and low expression of GLS2 in HCC correlated with survival time of HCC patients. Expression of GLS1 and GLS2 were independent indexes for survival time, although prognosis was predominantly determined by the level of GLS1 expression. These findings indicate that GLS1 expression is a sensitive and specific biomarker for pathological diagnosis and prognosis of HCC.

## RESULTS

### GLS1 expression is preferentially upregulated in HCC tumor cells and GLS2 is preferentially expressed in normal hepatocytes

**Figure 1:**
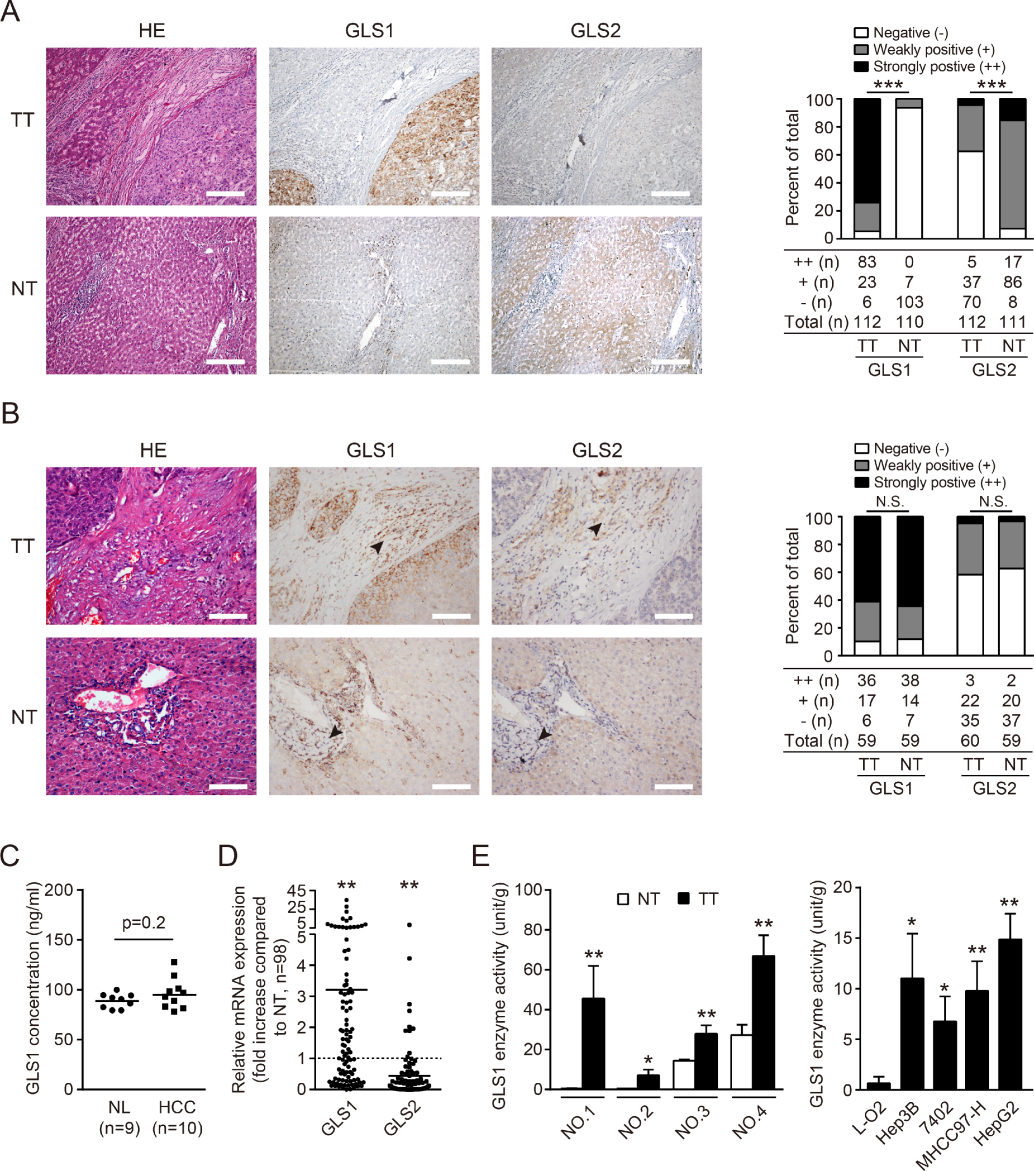
Expression and biodistribution of GLS1 and GLS2 in hepatocellular carcinoma **(A)** GLS1 and GLS2 were detected by immunohistochemical staining in 112 tumor tissues (TT) and paired non-tumor tissues (NT) from HCC patients. Representative staining of GLS1 and GLS2, and corresponding hematoxylin and eosin (HE) staining are shown (left panel). Bars = 200 μm. The expression and intensity of total samples were evaluated and classified into three grades: ++ strongly positive, + weakly positive; − negative (right panel). **(B)** The expression and distribution of GLS1and GLS2 in mesenchymal cells (black arrows) were evaluated by immunohistochemical staining in 60 paired TT and NT from HCC patients comprising a random subset of the 112 paired TT and NT samples from panel A. Representative staining of GLS1 and GLS2, and corresponding HE staining are shown (left panel). The expression and intensity of total specimens were evaluated (right panel). Bars = 100 μm. **(C)** GLS1 concentration in serum obtained from 9 normal healthy donors (NL) and 10 HCC patients was determined by ELISA. **(D)** Expression of GLS1 and GLS2 in 98 HCC tumors and paired adjacent non-tumor tissues was determined by quantitative RT-PCR. **(E)** Enzyme activity of GLS1 in TT and paired NT (*n* = 4, left panel), and in a non-malignant hepatic cell line (L-O2) and HCC cell lines (Hep3B, 7402, MHCC97-H, HepG2) (right panel); *p < 0.05, **p < 0.01, ***p < 0.001, N.S. not significant.

To investigate the expression and biodistribution of GLS1 and GLS2 in HCC, we performed immunohistochemical staining for GLS1 and GLS2 on the first group of paired tumor tissues (TT) and adjacent none tumor tissues (NT) from HCC patients. In the analysis of 112 cases, strongly positive staining against GLS1 in HCC tumor cells was observed in 83 cases (74.11%), weakly positive staining was found in 23 cases (20.54%), and negative staining was found in 6 cases (5.36%) (Figure 1A). Conversely, among adjacent NT hepatocytes from 110 cases analyzed, negative GLS1 staining was found in 103 cases (93.64%), and weakly positive staining was found in 7 cases (6.36%). These data suggest that GLS1 is highly expressed by HCC cells. We found negative staining for GLS2 in 70 of 112 HCC cases (62.5%), and positive staining in 103 of 111 cases in NT hepatocytes of NT (92.7%) (Figure 1A). Statistically, the expression of GLS1 in TT was significantly higher than in NT, while expression of GLS2 in TT was significantly lower than in NT. Additional immunohistochemical staining of GLS1 and GLS2 in TT and NT at a serial of magnifications is shown in Supplementary Figure 2A and 2B. We analyzed the available clinical information of the cases studied. We found that tumor capsule invaded was the only parameter showing clinical significance associated with differential expression of GLS1 and GLS2 (Supplementary Table 1). We found positive GLS1 staining in some mesenchymal cells in TT and NT. Among stained mesenchymal cells, no differences in expression intensity showed were observed. No GLS2 staining was observed in mesenchymal cells in TT or NT (Figure 1B). To determine if serum levels of GLS1 correlated with GLS1 staining of HCC cells, we analyzed sera from 10 HCC patients from group 1 and 9 healthy volunteers from group 2. GLS1 protein was detected a relative high levels in serum (about 100 ng/ml), but there was no difference between HCC patients and controls (Figure 1C).

To determine the gene expression pattern of GLS1, 98 HCCs from group 1 with paired TT and NT were analyzed by quantitative RT-PCR (Figure 1D). Expression of *GLS1* mRNA was significantly higher in TT than in NT. Again, expression of *GLS2* mRNA was significantly lower in TT than in paired NT (Figure 1D).

We also wanted to know if the enzymatic activity of GLS1 was elevated in HCC. To this end, we evaluated GLS1 enzymatic activity in 4 paired TT and NT samples from HCC patients. GLS1 activity was significantly higher in all TT samples than in NT samples (Figure 1E, left panel). Consistent with this, GLS1 activity was significantly higher in HCC cells lines than in a non-malignant hepatic cell line (Figure 1E, right panel).

Taken together, these results indicate that GLS2 is preferentially expressed in normal hepatocytes, but in HCC tumor cells GLS1 expression is upregulated and GLS2 expression is downregulated.

### GLS1 expression possesses high sensitivity and specificity for hepatocellular carcinoma

**Figure 2:**
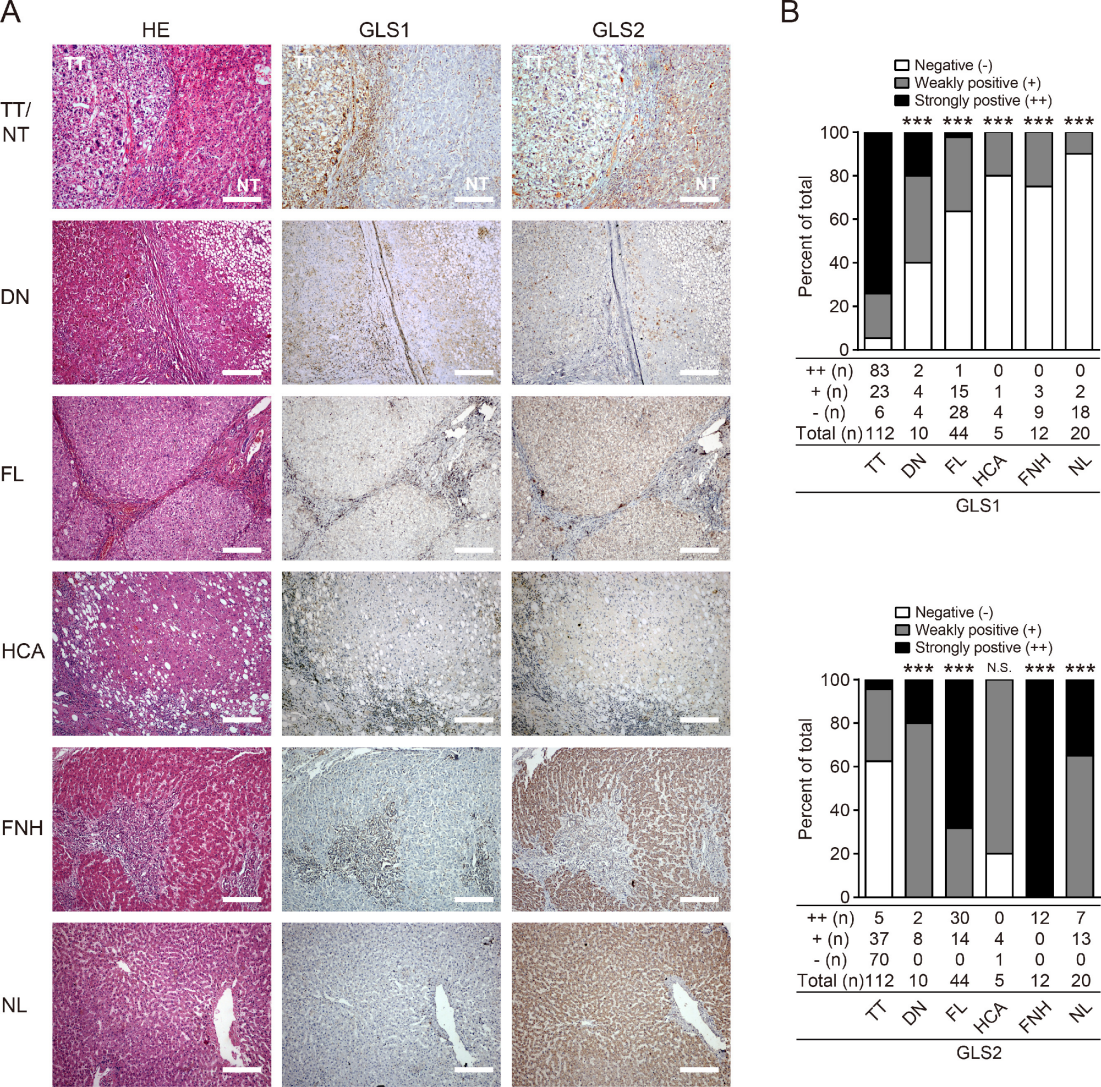
Expression and biodistribution of GLS1 and GLS2 in HCC and other liver diseases **(A)** HE and immunohistochemical staining for GLS1 and GLS2 in HCC tumor tissues (TT, *n* = 112), dysplastic nodule tissues (DN, *n* = 10), fibrotic liver tissues (FL, *n* = 44), hepatocellular adenoma tissues (HCA, *n* = 5), focal nodule hyperplastic tissues (FNH, *n* = 12), and normal liver tissues (NL, *n* = 20). Bars = 200 μm. **(B)** Quantitation of expression intensity and frequency of GLS1 (upper panel) and GLS2 (lower panel). Intensity was categorized into three grades: negative (−), weakly positive (+), and strongly positive (++); ***p < 0.001, N.S. not significant.

We wanted to determine the specificity and sensitivity of GLS1 as a marker for HCC. To this end, the expression and distribution of GLS1 and GLS2 were determined in a serial set of liver tissues including 20 normal liver (NL), 44 fibrotic liver (FL), 12 focal nodular hyperplasias (FNH), 5 hepatocellular adenoma (HCA), and 10 dysplastic nodules (DN) and compared to expression in 112 HCC samples. The histological features and immunohistochemical staining of GLS1 and GLS2 in these tissues are shown in Figure 2A and 2B and Supplementary Figure 2. Staining for GLS1 was positive in 94.64% of HCC samples (83/112 were strongly stained and 23/112 were weakly stained), 60% of DN (2/10 were strongly stained and 4/10 were weakly stained), 36.4% of fibrotic liver (1/44 was strongly stained and 15/44 were weakly stained), 20% of HCA (1/5 was weakly stained), 25% of FNH (3/12 were weakly stained), and 10% of normal liver samples (2/20 were weakly stained). We found that both positivity and intensity of GLS1 in HCC were significantly higher than in other liver diseases or normal liver tissues (*p* < 0.001, Figure 2A and 2B upper panel). Interestingly, GLS1 positivity in DN, a premalignant liver disease, was also significantly higher than in NL (*p* = 0.010) and FL (*p* = 0.069) (Figure 2B upper panel). We also evaluated the expression of GLS2 in these specimens. Similar to our previous observations, both positivity and intensity of GLS2 expression were significantly lower in HCC compared to DN, FL, FNH, and NL (*p* < 0.001, Figure 2A and 2B lower panel). Staining for GLS2 was positive in 37.5% of HCC samples (5/112 were strongly stained and 37/112 were weakly stained) and 80% or 100% in other liver diseases and normal liver tissue (Figure 2B lower panel).

Generally, when the threshold for GLS1 positivity was set at strongly stained (++, strongly positive), GLS1 was 74.11% (83/112) in HCC, the sensitivity and specificity of GLS1 for HCC was 83/86 (96.51%) and 88/117 (75.21%), respectively, among the tissues including HCC, HCA, DN, FL and NL (Table 1). Interestingly, the sensitivity for HCC could be increased up to 100% when tissue staining was both GLS2 negative (−) and GLS1 strongly positive (++).

**Table 1:** GLS1 and GLS2 expression in a serial set of liver tissues and their sensitivity and specificity for HCC

Antigen	Grade	TT	DN	FL	HCA	FNH	NL	Total	Positive (%)	Negative (%)	Sensitivity (%)	Specificity (%)
GLS1	−	6	4	28	4	9	18	69	74.1% (83/112)	25.9% (29/112)	96.5% (83/86)	75.2% (1–29/117)
	+	23	4	15	1	3	2	48				
	++	83	2	1	0	0	0	86				
	Total	112	10	44	5	12	20	203				
GLS2	−	70	0	0	1	0	0	71	62.5% (70/112)	37.5% (42/112)	98.6% (70/71)	68.2% (1–42/132)
	+	37	8	14	4	0	13	76				
	++	5	2	30	0	12	7	56				
	Total	112	10	44	5	12	20	203				
GLS1 (++) or GLS2 (−)		108	2	1	1	0	0	112	96.4% (108/112)	3.6% (4/112)	96.4% (108/112)	95.6% (1–4/91)
Total		112	10	44	5	12	20	203				
GLS1 (++) and GLS2 (−)		45	0	0	0	0	0	45	40.2% (45/112)	59.8% (67/112)	100% (45/45)	57.6% (1–67/158)
Total		112	10	44	5	12	20	203				

TT, hepatocellular carcinoma; NT, adjacent non-tumor tissues; HCA, hepatocellular adenoma; DN, dysplastic nodules; FNH, focal nodular hyperplasia; FL, fibrotic liver; NL, normal liver.

Intensity of GLS1 and GLS2 was evaluated and scored: ++, strongly positive; +, weakly positive; −, negative.

Taken together, these data suggest that tissue expression of GLS1 may provide a specific marker that will be useful in HCC diagnosis.

### Validation of sensitivity and specificity of GLS1 for hepatocellular carcinoma

**Figure 3:**
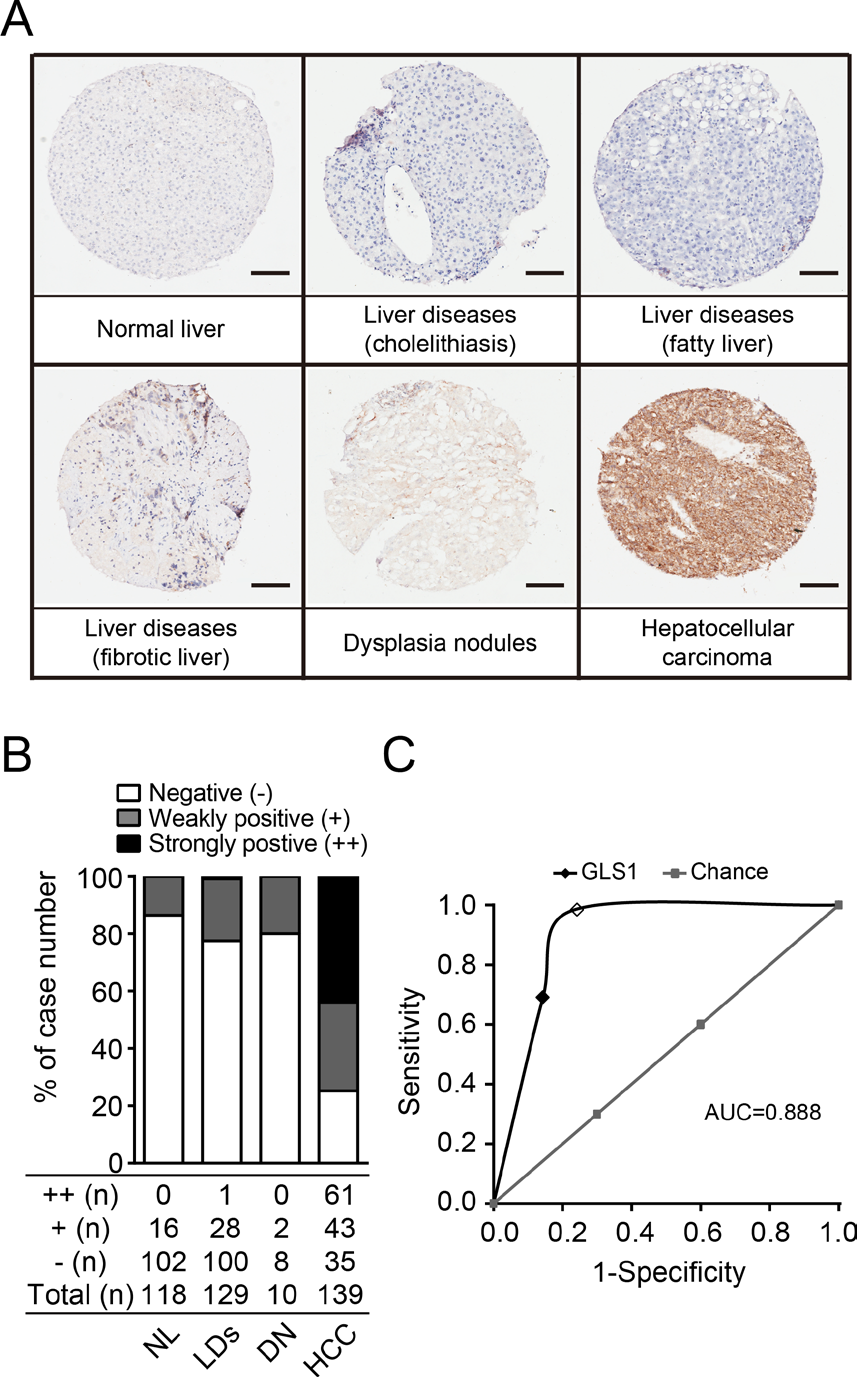
Tissue microarray analysis validates the sensitivity and specificity of GLS1 for diagnosis of HCC **(A)** The expression of GLS1 was determined by immunohistochemical staining on tissue microarrays. Representative staining of each pathological category is presented. Bars = 100 μm. **(B)** Quantitation of expression intensity and frequency of GLS1 in all tissue punches. LDs, liver diseases, including cholelithiasis, fatty liver and fibrotic liver. **(C)** Sensitivity and specificity of GLS1 for HCC analyzed by ROC curve (dark line). Positive GLS1 staining in HCC was counted by two thresholds: strongly stained (open dot) and weakly stained (filled dot). The AUC value is 0.888.

Having shown that GLS1 possesses the sensitivity and specificity for pathologic diagnosis of HCC, we sought to validate this finding by tissue microarray (TMA, OD-CT-DgLiv01 with 478 spots) analysis. The liver tissues were confirmed and categorized into normal liver, dysplasia nodule, HCC and other liver diseases. We found that GLS1 was negatively stained in hepatocytes of normal liver, and cholelithiasis, weakly stained in tissues of fatty liver, fibrotic liver, and dysplasia nodules, and strongly stained in tumor cells of HCC (Figure 3A). The frequency and intensity of GLS1 staining are summarized in Figure 3B. The intensity of GLS1 staining in HCC was much higher than in any other tissue. When the cut-off for positivity for GLS1 was set at strongly positive (++) staining intensity, the senstivity and specificity of GLS1 for HCC was 98.39% and 76.64%. If the cut-off for positivity for GLS1 was set at weakly positive (+) staining intensity, the senstivity was decreased to 76.65% while the specificity was increased to 85.71%. The AUC (area under the receiver operating characteristic curve) value was up to 0.888 (Figure 3C). These results provide validation for our proposed use of GLS1 as a sensitive and specific diagnostic marker for HCC.

### Glutamine metabolism is switched from GLS2 to GLS1 during hepatic malignant progression towards HCC

**Figure 4:**
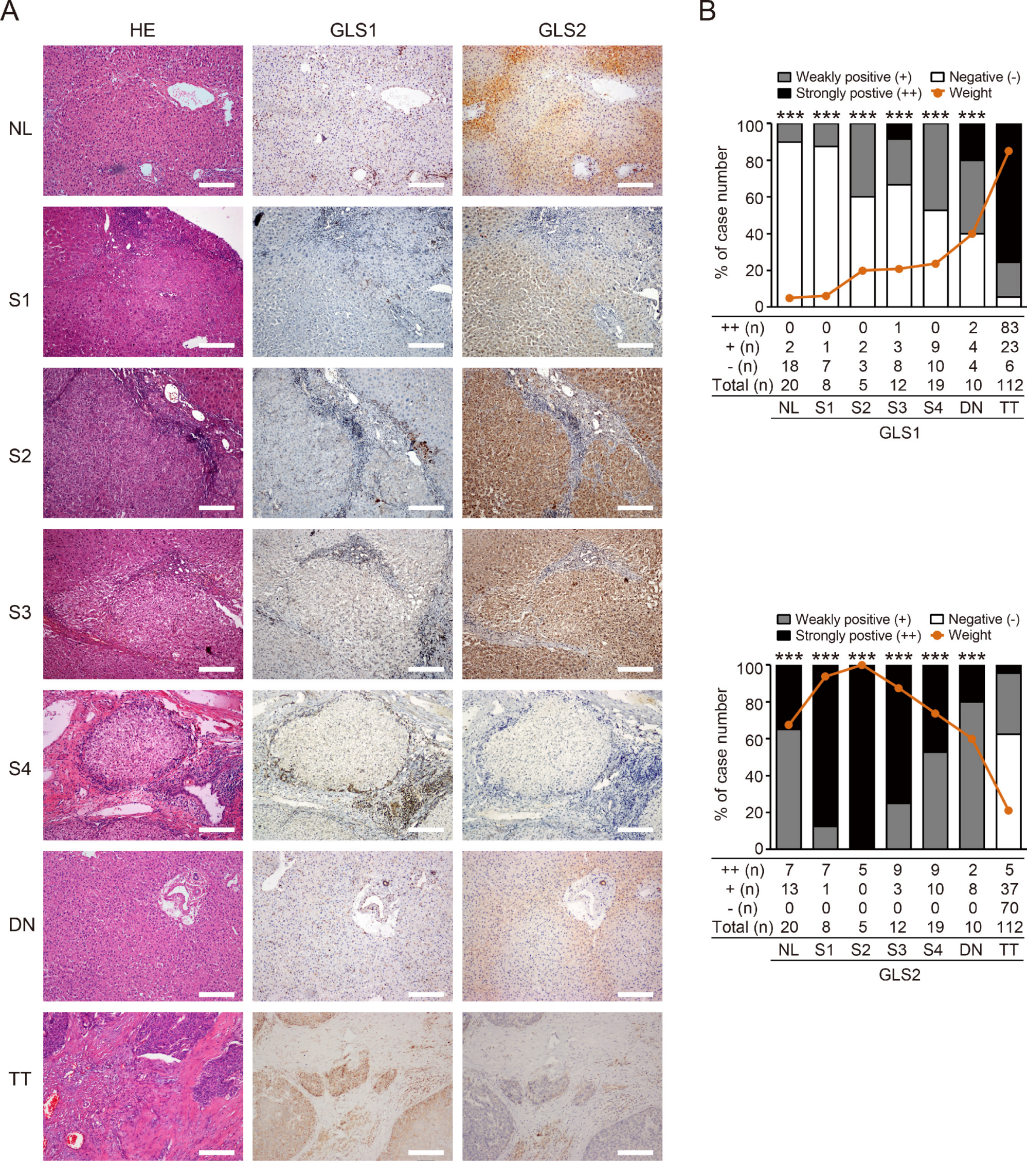
Glutamine metabolism is switched from GLS2 to GLS1 during oncogenic transformation to HCC **(A)** HE and immunohistochemical staining for GLS1 and GLS2 in normal liver tissues (NL, *n* = 20), fibrotic liver tissues from Grade I to V (S1–S4, *n* = 44), dysplastic nodule tissues (DN, *n* = 10), and HCC tumor tissues (TT, *n* = 112). Staining of representative sections are shown. Bars = 200 μm. **(B)** Quantitation of intensity and frequency of GLS1 (upper panel) and GLS2 (lower panel) expression. The intensity was scored as 2 (strongly positive), 1 (weakly positive), or 0 (negative). Brown lines indicate aggregate positivity in different tissues, calculated according to the frequency and intensity: ∑ Frequency × Intensity/2. ***p < 0.001.

It has been shown that glutamine metabolism switches from GLS2 to GLS1 in MYC-induced mouse liver cancer [21]. It is unknown whether this metabolic switch also plays a role in human liver oncogenic transformation. To address this possibility, we evaluated the expression of GLS1 and GLS2 in a serial set of liver tissues mimicking HCC transformation; these included normal liver, fibrotic liver tissues from Grade I to IV, dysplasia nodule, and HCC tissues. The histological features and the immunohistochemical staining of GLS1 and GLS2 of fibrotic liver from grade I to IV are shown in Supplementary Figure 2 (D–G). GLS1 expression was low in normal liver tissues but was progressively upregulated in parallel with disease progression, and was high in HCC (*p* < 0.001, Figure 4A and 4B upper panel). Conversely, the intensity of GLS2 expression was high in normal liver and low-grade fibrotic liver tissues, and was low in HCC (*p* < 0.001, Figure 4A and 4B lower panel). We also investigated the correlation of *GLS1* with *c-MYC* at gene level, and found that the expression of *GLS1* in TT was associated with *c-MYC* expression (Supplementary Figure 3). These results suggest that glutamine metabolism may be switched from GLS2 to GLS1 during human HCC transformation.

### GLS1 and GLS2 are prognostic markers for HCC patients

**Figure 5:**
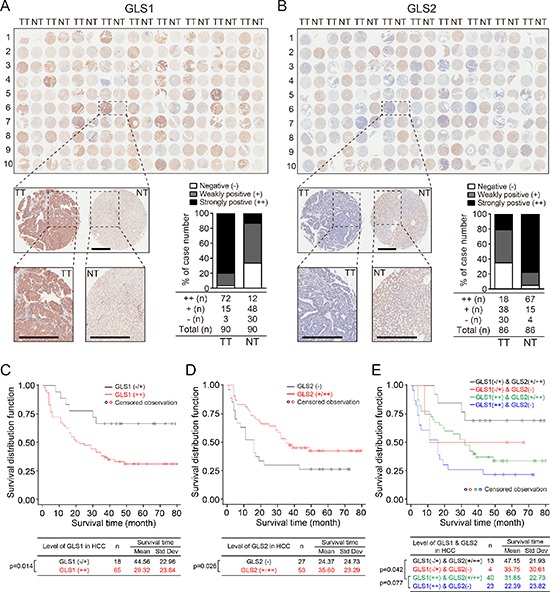
Expression of GLS1and GLS2 correlate with survival times of HCC patients **(A** and **B)** Expression of GLS1 and GLS2 determined by immunohistochemical staining in tissue arrays containing tumor and paired adjacent non-tumor tissues from 90 HCC patients. Insets show higher magnifications of stainings of one set of paired tissues. Bars = 500 μm. Histograms show quantitation of expression and intensity of staining of all samples. Intensity of staining was classified into three grades: ++ strongly positive, + weakly positive, and − negative. **(C** and **D)** Survival curves of 90 HCC patients divided into 2 groups according to staining intensity. (C) GLS1 survival curves. Black line: low/non-expression (−/+); red line: high-expression (++). (D) GLS2 survival curves. Black line: non-expression (−); red line: expression (+/++). Kaplan–Meier survival curves were based on time to death from disease or on time to biochemical recurrence (BCR). **(E)** Survival curves of 90 HCC patients based on combined GLS1 and GLS2 expression. Black line: GLS1 −/+ and GLS2 +/++ (*n* = 13); red line: GLS1 −/+ and GLS2 − (*n* = 4); green line: GLS1 ++ and GLS2 +/++ (*n* = 40); blue line: GLS1 ++ and GLS2 − (*n* = 23). Kaplan–Meier survival curve based on time to death from disease or on time to biochemical recurrence (BCR).

Finally, we sought to determine if expression of GLS1 and GLS2 could be predictive biomarkers for clinical outcomes in HCC patients. To this end, we examined the expression of GLS1 and GLS2 in TMA (HLiv-HCC180Sur-01) with 90 paired NT and TT obtained from HCC patients, and analyzed the relationship between GLS1/GLS2 expression and patient survival. In line with our previous observation, immunoreactivity against GLS1 was observed primarily in the cytoplasm of HCC cells, and was strongly positive (++) in about 80% of HCC (72 of 90 cases), weak (+) in 16.67% (15/90), and was absent in 3.33% (3/90) cases. In paired adjacent non-tumor tissues, GLS1 immunoreactivity was absent in 33.3% (30/90) of cases, weak in 53.3% (48/90) of cases and strong in 13.3% (12/90) of cases (Figure 5A). Intensive GLS2 staining (++) was observed in 77.9% (67/86) of adjacent non-tumor tissues, and was observed in 20.9% (18/86) of tumor tissues (Figure 5B). Patients with high GLS1 expression (++) had a markedly shorter overall survival time (29.32 months vs. 44.56 months) compared to patients with absent or low GLS1 expression (−/+) (Figure 5C). We also evaluated the relationship between expression of GLS2 in tumor tissues and survival time. We found that the patients exhibiting GLS2 expression (+/++) in tumor tissues had a significantly prolonged survival time (35.60 months vs. 24.37 months) compared to patients without tumor GLS2 expression (Figure 5D). Consistently, patients with high tumor GLS1 but no tumoral GLS2 exhibited the shortest survival times (22.39 months), whereas patients with tumoral GLS2 (+/++) and low tumoral GLS1 (−/+) had significantly prolonged survival times (47.15 months). Patients with tumoral GLS2 (+/++) and high tumoral GLS1 (++) exhibited median survival times (31.85 months) (Figure 5E). Multivariate survival analysis showed that the scales for both GLS1 and GLS2 were independent indexes for survival time of HCC patients (*p* = 0.003). The clinical information of 90 HCC cases are summarized in Supplementary Table 2. We found that the expression of GLS1 correlated with age, while no correlation was found between GLS1 and gender, morphology, tumor number, clinical stage of the tumor (TNM), tumor size, or volume. Taken together, our results show that GLS1 and GLS2 are potential prognostic biomarkers for HCC.

## DISCUSSION

HCC occurs mainly in patients with progressive liver disease. Sensitive and specific pathological biomarkers to distinguish HCC from cirrhotic or dysplastic nodules are not available. Here, we show that GLS1 was highly expressed in HCC, and is a sensitive and specific marker for HCC. GLS2 was mainly expressed in non-tumor hepatocytes, and there was a metabolic switch from GLS2 to GLS1 in HCC. While both GLS1 and GLS2 were independent indexes for survival time, prognosis was predominantly determined by GLS1 expression. This investigation of a large number of human HCC specimens confirms that GLS1 is a potential biomarker for HCC pathological diagnosis and prognosis.

Stromal invasion is considered to be the distinguishing diagnostic criterion for differentiating early HCC from high-grade dysplastic nodules (HGDN). However, it is difficult to recognize stromal invasion by histopathological analysis of needle biopsies because included portal tracts may be absent. Therefore, other diagnostic criteria are necessary. Although prospective studies indicate some clinical usefulness of a panel of markers including GPC3, HSP70, and GS for the diagnosis of early HCC, analysis of this panel only slightly increases the diagnostic accuracy [2]. The biodistribution pattern of GS is a commonly used marker for clinical pathological diagnosis of HCC; homogeneous distribution of GS indicates HCC, whereas diffuse distribution of GS is commonly found in normal liver tissues [22]. We found that there was no difference in either expression intensity or frequency of GS between tumor cells and normal liver tissues (Supplementary Figure 4). Thus, GS might not be an optimal biomarker despite its common use in clinical diagnosis of HCC. We found that both GLS1 expression intensity and frequency, as well as enzyme activity were highly upregulated in HCC tumor tissues compared to non-tumor tissues. Sensitivity and specificity of GLS1 for HCC were 96.51% and 75.21%, respectively. These results indicate that GLS1 is a specific and sensitive biomarker that will likely improve pathologic diagnosis of HCC.

Although GLS1 staining was mainly negative in non-tumor tissues, a small portion of normal liver mesenchymal cells were GLS1 positive; however, it is easy to distinguish mesenchymal cells from hepatocytes by morphology and histological distribution. Thus, this staining of a minor population of normal cells is not likely to obscure the pathological diagnosis of HCC. Of note, when results of GLS1 and GLS2 staining in tumor tissues were combined, the sensitivity of these markers for HCC pathological diagnosis reached 100%. These results suggest the possibility using both markers (GLS1 and GLS2) for HCC diagnosis in difficult cases.

In a serial of human liver diseases that mimic the progression of HCC transformation, i.e., from normal liver, to fibrotic liver, to dysplastic nodule, to HCC, we found increased GLS1 expression at each step, while GLS2 was significantly down-regulated in HCC. The mechanisms of GLS2-to-GLS1 switch in HCC remain to be clarified. A recent study showed that glutamine metabolism was increased in MYC-induced mouse liver tumors, and that glutamine metabolism switched from GLS2 to GLS1 [21]. We also confirmed that the expression of *GLS1* correlated with *c-MYC* expression. *GLS2* has been considered to be a tumor suppressor gene, whereas *GLS1* is considered to be an oncogene [13]. Thus, the metabolic switch from GLS2 to GLS1 might be indispensable for HCC oncogenic transformation. One plausible possibility is that GLS1 might be crucial for liver malignant progression simply due to its higher hydrolytic efficiency than GLS2 [23]. In line with this, we found that HCC patients who exhibited high GLS1 expression suffered decreased survival, and that patients who exhibited high GLS2 expression in tumor tissues survived longer.

To date, there is no optimal predictive marker for HCC. We showed that both GLS1 and GLS2 were independent indexes for HCC prognosis. High GLS1 and/or low GLS2 in HCC predicted a poor outcome. Although our data support the view that GLS1 is a dominant predictive marker, GLS2 expression within tumor tissues may provide more precise survival information for patients with HCC. For example, the poorest prognosis was seen in patients in whom high GLS1 expression in HCC cells was accompanied by loss of GLS2 expression. It is possible that HCC with higher intensity of GLS1 might be metabolically more active and this may contribute to increased mortality. A previous research has shown the synergistic role of GLS1 silencing and GLS2 overexpression in apoptosis, antioxidant status and cellular motility in glioma cells [24]. It remains to be determined whether upregulation of GLS1 in HCC is associated with other malignant phenotypes such as cell migration, invasion, tumor neovascularization, or epithelial to mesenchymal transition.

As targeting glutaminase activity has been shown to inhibit oncogenic transformation [25], this study and others suggest GLS1 as a potential target for cancer therapy [26–28]. It might be possible to use GLS1 expression in HCC as a biomarker for tailored antitumor therapy targeting altered glutaminolysis. A recent article demonstrate that GLS2 plays an important role in tumor suppression in HCC, and that negative regulation of PI3K/AKT signaling contributes greatly to this function of GLS2 [29].

In this retrospective study, we have shown that GLS1 is a potential pathological biomarker for HCC diagnosis and prognosis. Retrospective and prospective clinic studies are ongoing in multiple centers to further validate the sensitivity and specificity of GLS1 and GLS2 in HCC.

## MATERIALS AND METHODS

### Patients and tissue specimens

Four groups of tissues from HCC, normal liver, and from other liver diseases were enrolled. The tissue groups and their corresponding applications are summarized in Supplementary Figure 1. The protocol was approved by the research ethics committee of Drum Tower hospital. Informed consent in writing was obtained from each patient, and the study protocol conformed to the ethical guidelines of the 1975 Declaration of Helsinki as reflected in a prior approval by the appropriate institutional review committee.

The first group of tissues (group 1) were paired tumor and adjacent non-tumor tissues from 112 patients with hepatocellular carcinoma (HCC) undergoing partial hepatectomy. These patients were enrolled in a study in the Department of Hepatobiliary Surgery of Drum Tower Hospital between January 2004 and August 2010. There were 94 males (83.93%), and the mean age was 52.95 ± 10.19 years. Patients were followed for a median time of 34 (18–46) months. None of the patients had received preoperative treatment. HCC diagnosis was based on guidelines of Bruix and Sherman [30]. Tumor differentiation was defined according to the Edmondson grading system [31]. Serial sections of HCC tumors containing adjacent non-tumor tissues were examined to identify tumor encapsulation, microscopic venous invasion, and microsatellite lesions. The degree of HCC invasiveness was verified according to the invasiveness scoring system for HCC [32, 33]. Areas of tissue necrosis and hemorrhage were excluded. All tissue samples were snap frozen immediately after resection, and kept in liquid nitrogen until they were used for experiments. Before surgery, peripheral blood of each patient was collected in EDTA-containing tubes for plasma preparation.

The second group of tissues (group 2) included 5 hepatocellular adenomas (HCA), 10 dysplastic nodules (DN), 12 focal nodular hyperplasias (FNH), 44 fibrotic liver (FL), and 22 normal liver (NL) samples from living donors or donations after cardiac death of donors. These specimens were obtained from patients who underwent surgery at the Drum Tower Hospital during 2005 to 2013. Peripheral blood samples were obtained from 20 healthy male volunteers aged 38 to 59 years.

All histopathology and immunostaining was evaluated by a senior pathologist, who was unaware of the preoperative clinical data.

### Tissue microarray

A third group of tissues on a commercially available tissue microarray (TMA) (Shanghai Biochip Company Ltd, Shanghai, China, OD-CT-DgLiv01) was used to validate sensitivity and specificity of GLS1 for HCC. The analyzed tissue array contained 478 spots including HCC tumor and paired non-tumor tissues, and other tissues from normal liver and other liver diseases including cholelithiasis, fatty liver, fibrotic liver, and dysplasia nodule.

A fourth group of tissues on a commercially available microarray (Shanghai Biochip Company Ltd, Shanghai, China, HLiv-HCC180Sur-01), which included 90 paired TT and NT of HCC, was used to analyze the relationship between GLS1 expression and the biological behaviors of HCC patients. Details of the array are available on the websitehttp://www.outdobiotech.com/en/product-detail-212.html. Human specimens presented on these microarrays were approved by proper institutional review boards.

### Immunohistochemistry of paraffin-embedded sections and tissue microarray

Specimens were paraffin-embedded. Serial 4 μm sections were cut, deparaffinized, blocked, and incubated at 4°C overnight with the primary antibody, followed by horseradish peroxidase-labeled secondary antibody. Positive binding was visualized with diaminobenzidine solution followed by counterstaining with hematoxylin. Negative controls were achieved by substituting the primary antibodies with PBS. Immunohistochemistry was performed using a SuperPictureTM 3rd Gen IHC Detection Kit (Zymed Laboratories, San Francisco, CA; #87 - 8973) according to the manufacturer's recommendations. The primary antibodies used in this study were: rabbit monoclonal antibody to GLS1 (recognizes to both KGA and GAC isoforms, 1:3200, Epitomics, Burlingame, CA, #7485-1), rabbit polyclonal antibody to GLS2 (recognizes to both GAB and LGA isoforms, 1:400, Abcam, Hong Kong, China, #ab113509), and mouse monoclonal antibody to glutamine synthetase (GS, 1:300, Abcam, Hong Kong, China, #ab64613). Primary antibodies were detected with an EnVision+/HRP, Rabbit (AEC+) kit (K4008, Dako, Glostrup, Denmark).

For immunohistochemical analysis of GLS1 on TMAs, heat-induced antigen-retrieval procedures were performed, after which the primary antibody (Anti-human GLS1, 1:6400, Epitomics, Burlingame, CA, #7485-1) was incubated at 48°C for 18 hours. Biotinylated secondary antibody was incubated for 30 min, and binding was detected with avidin-biotin-HRP complex (Vectastain ABC kit; Vector Laboratories, Burlingame, CA).

The expression of GLS1/GLS2 was analyzed by extent of staining according to the Fromowitz standard [34]. In brief, five randomized visual fields were observed and photographed. Scores representing the numbers of cells stained (range score) were graded according to the percent of positively stained cells: 0–5%, 5–50%, and > 50%. Scores representing the intensity of staining (extent score) were “−” (unstained, negative), “+” (brown stained, weakly positive), or “++” (dark brown stained, strongly positive). Thus, expression status of the target protein was evaluated jointly by range and extent scores.

### Cell lines

The MHCC97-H human HCC cell line with high metastatic potential was obtained from the Liver Cancer Institute, Zhongshan Hospital, Fudan University, Shanghai, China [35]. Human normal liver cell line (L-O2), human hepatocellular carcinoma cell lines (Hep3B, 7402, and HepG2) were obtained from American Type Culture Collection. All cell lines were routinely maintained at 37°C and 5% CO2 in DMEM supplemented with fetal bovine serum.

### Quantitative RT-PCR

HCC cell lines, normal liver tissues from the second group, HCC samples and their paired non-tumor liver tissues randomly selected from the first group were analyzed by quantitative RT-PCR as described [36] with slight modification. Briefly, 2 μg total RNA were reverse-transcribed (TaKaRa, Shiga, Japan, DRR036A), and gene expression was quantified using the FastStart Universal SYBR Green Master (Roche, Mannheim, Germany, 04913914001) by the Real-Time PCR system (Applied Biosystems ViiA™ 7 Real-Time PCR System, Foster, CA). Gene expression was calculated with the comparative Ct method and normalized to the endogenous levels of GAPDH. All experiments were performed in triplicate. Genes and their primer sequences used for qRT-PCR were as follows: *GAPDH,* CCATGTTCGTCATGGGTGT GAACCA and GCCAGTAGAGGCAGGGATGATGTTC; *GLS1* (KGA), GTCACGATCTTGTTTCTCT GTG and GTCCAAAGAGCAGTGCTTCATCC ATG; *GLS2* (GAB and LGA), TGCCTATAGTGGC GATGTCTCA and GTTCCATATCCATGGC TGACAA; *MYC,* CTTCTCTCCGTCCTCGG ATTCT and GAAGGTGATCCAGACTCTG ACCTT; *GLUL*, CCTGCTTGTATGCTGGAGTC and GATCTCCCATGCTGATTCCT.

### GLS enzyme activity assay

Cellular glutaminase enzyme activity was analyzed using Cellular/Tissue Glutaminase Activity Assay Kits (GMS50374.1/GMS50374.2, Genmed Scientifics Inc., MA) according to the manufacturer's protocol. Briefly, cells or tissue were lysed with lysis buffer on ice. Supernatant was harvested and incubated with reaction buffer at 37°C for 30 minutes followed by stop solution (marked as solution A). The reaction substrate, enzymatic solution, and buffer were mixed at 37°C for 3 minutes prior to incubation with solution A at 37°C for 30 min. The absorbance was monitored by a spectrophotometer at 340 nm. Enzyme activity was calculated according to the kit manufacturer's supplied formula.

### ELISA assay

Sera and cell supernatants were harvested, centrifuged, and processed for ELISA assay using a kidney-type glutaminase ELISA kit (Cloud-Clone Corp., Houston, TX, #SEJ026Hu) according to the manufacturer's instruction.

### Statistical analysis

For comparisons, the chi-squared test, Fisher's exact test, one-way analysis of variance, and two-tailed Student *t*-test were performed as appropriate. Correlations were analyzed by Spearman Rank-Order method. The discriminatory power for the putative marker was further described via receiver operating characteristic (ROC) area under the curve (AUC) analysis. Univariate or multivariate survival analysis was assessed with LIFETEST or PHREG method. Multivariate regression models were fitted to identify independent factors related to overall and tumor-specific mortality adjusted for competing risk to die for other causes; only variables with *p* < 0.2 were retained for multivariate analysis. Results were expressed as hazard ratio (HR) with 95% CI. *p* < 0.05 was considered to be statistically significant. All tests were two sided. Data analysis was done with SAS Version 12.0 software (SPSS Inc., Chicago, IL).

## SUPPLEMENTARY FIGURES AND TABLES



## Abbreviations

AUC: area under the receiver operating characteristic curve
DN: dysplastic nodules
FNH: focal nodular hyperplasia
FL: fibrotic liver
Gln: glutamine
GLS: glutaminase
GS: glutamine synthetase
HCA: hepatocellular adenoma
HCC: hepatocellular carcinoma
HE: hematoxylin and eosin
HR: hazard ratio
NL: normal liver
NT: non-tumor tissues
ROC: receiver operating characteristic
ROS: reactive oxygen species
TMA: tissue microarray
TT: tumor tissues
