# Supplementary material for: Kidney-type glutaminase (GLS1) is a biomarker for pathologic diagnosis and prognosis of hepatocellular carcinoma
Source: Oncotarget. 2015 Mar 26;6(10):7619–31. doi: 10.18632/oncotarget.3196 (PMC4480704; doi:10.18632/oncotarget.3196)
Supplement: Supplementary file 1 [file oncotarget-06-7619-s001.pdf]

SUPPLEMENTARY FIGURES AND TABLES

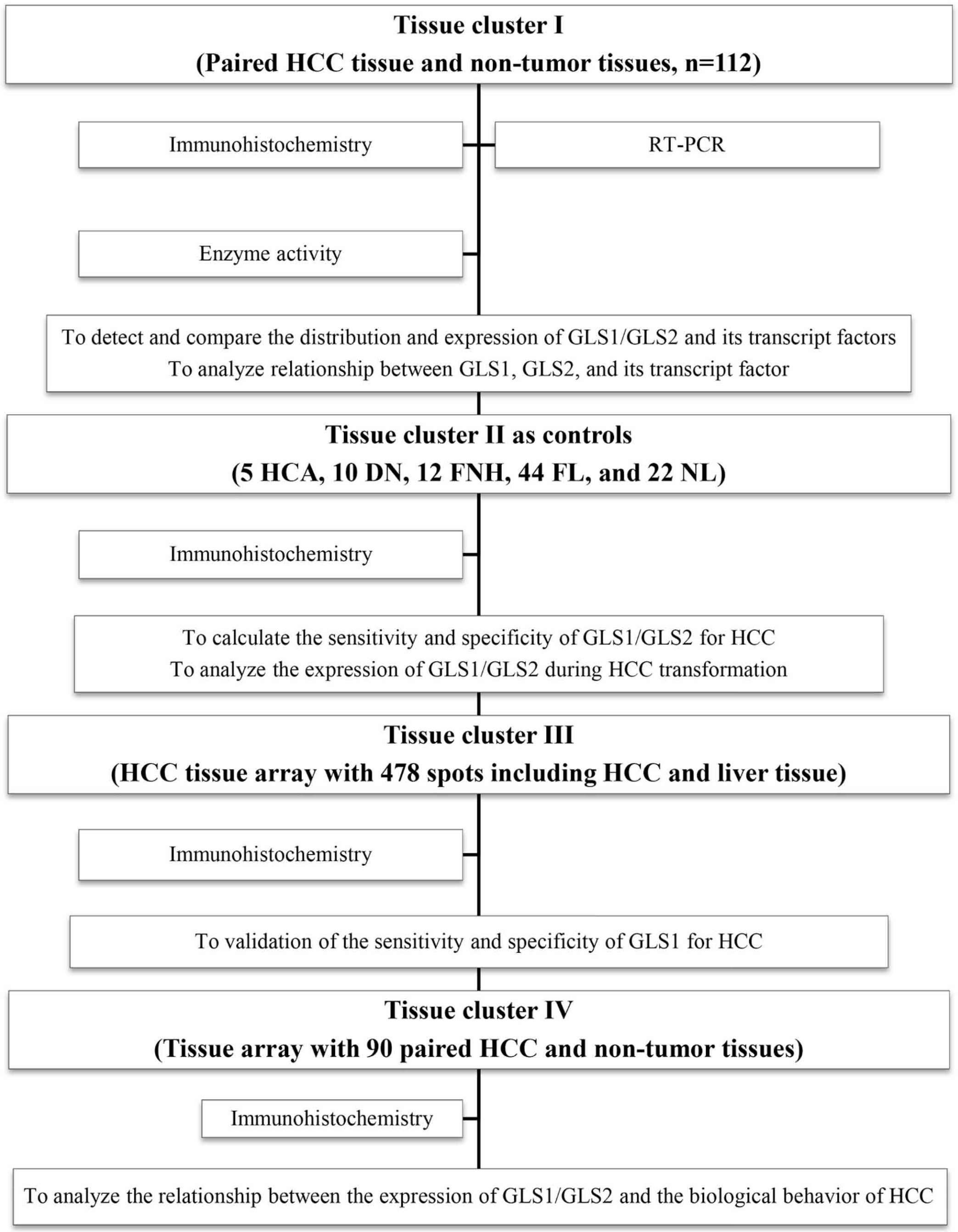

Supplementary Figure 1: Flow chart summarizing the tested samples. Firstly, the expression and biodistribution of GLS1/GLS2 in tumor tissue (TT) was compared with the adjacent non-tumor tissue (NT), and the relationship between GLS1/GLS2 and its transcript factor was analyzed in the tissue cluster I, 112 paired TT and NT obtained from HCC patients. Secondly, the sensitivity and specificity of GLS1 for diagnosis of HCC was evaluated, and HCC oncogenic transformation was analyzed in tissue cluster II, including 5 hepatocellular adenoma (HCA), 10 dysplastic nodules (DN), 12 focal nodular hyperplasia (FNH), 44 fibrotic liver (FL), and 22 normal liver (NL). Thirdly, the sensitivity and specificity of GLS1 was validated for HCC diagnosis in tissue cluster III with tissue array, including the paired tumor tissues and non-tumor tissues from 221 patients with HCC, and other tissues from healthy subjects to other liver diseases. Fourthly, the relationship between the expression of GLS1/GLS2 and the biological behavior of HCC was analyzed in tissue cluster IV with tissue array, including 90 paired tumor tissues (TT) and adjacent non-tumor tissues (NT).

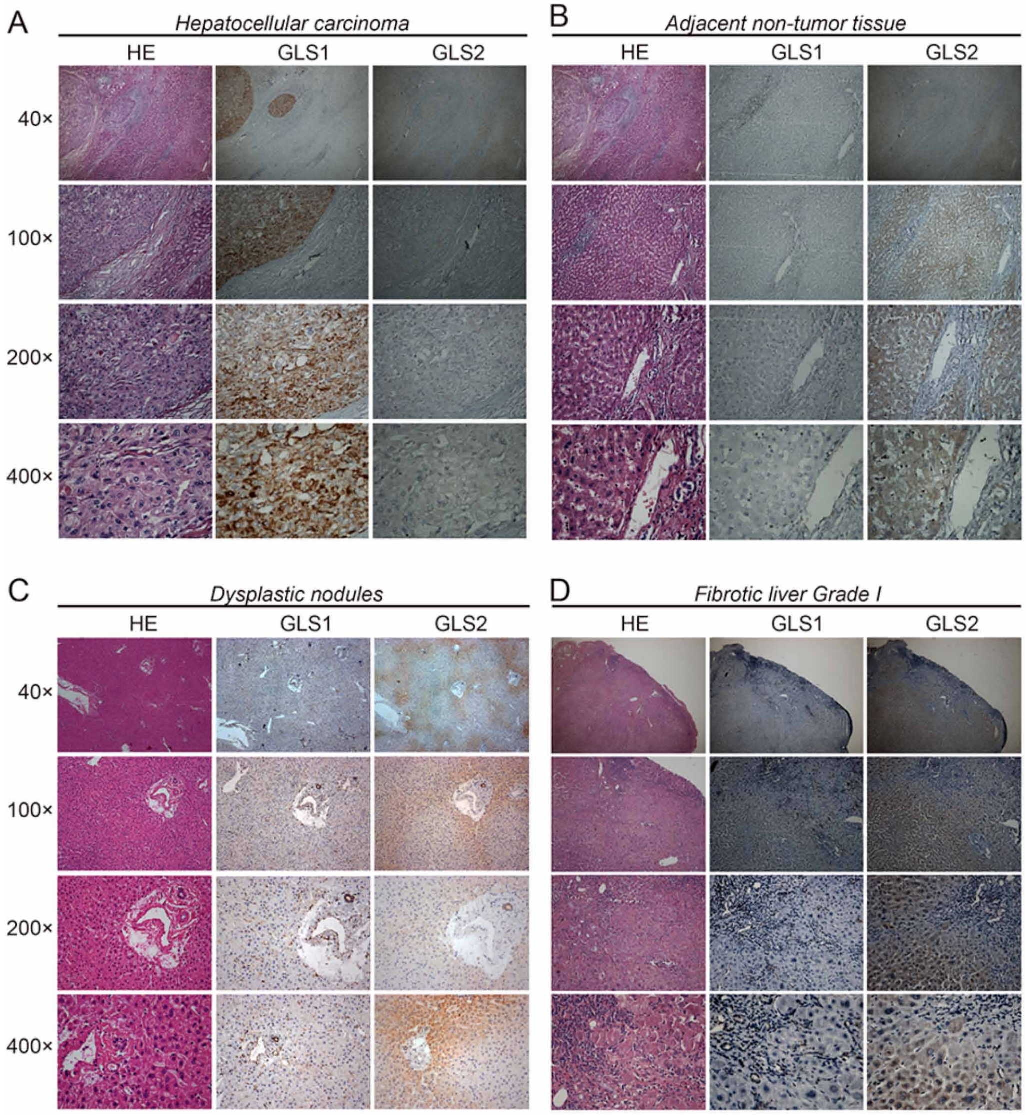

Supplementary Figure 2: Histological features and immunostaining of GLS1 and GLS2 in different type of liver diseases. The expression and distribution of GLS1/GLS2 were detected in a serial of liver diseases, including HCC **(A)** and the paired adjacent non-tumor tissue **(B)**, DN **(C)**, fibrotic liver from Grade I to IV **(D–G)**.

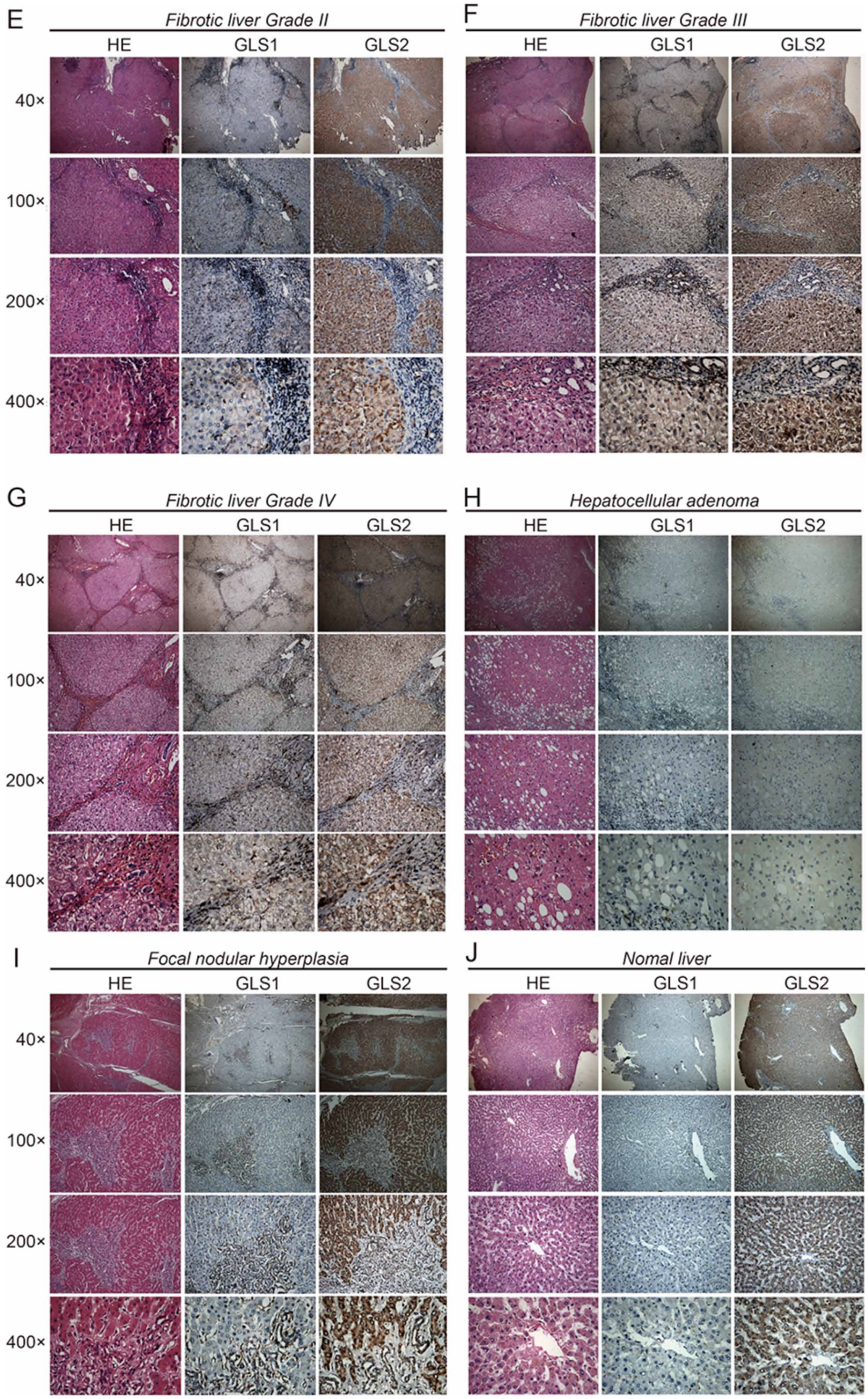

Supplementary Figure 2: (*Continued*) Histological features and immunostaining of GLS1 and GLS2 in different type of liver diseases. (D–G), HCA **(H)**, FNH **(I)**, normal liver **(J)** The representative staining of GLS1 and GLS2, and their corresponding hematoxylin and eosin (HE) staining are shown at magnifications as indicated.

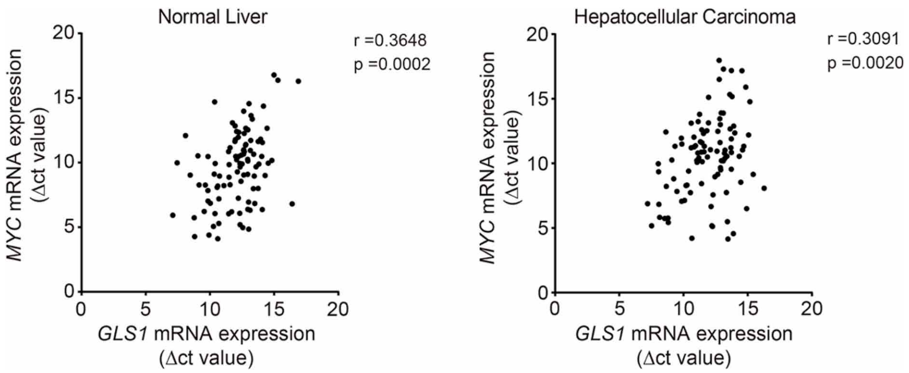

Supplementary Figure 3: Correlation of expression of GLS1 and MYC. Expressive associations between *GLS1* mRNA and *MYC* mRNA in normal liver (NL, left panel) or HCC (right panel) were evaluated using nonparametric Spearman's rank correlation analysis ($r = 0.3648$, $p = 0.0002$; $r = 0.3091$, $p = 0.0020$).

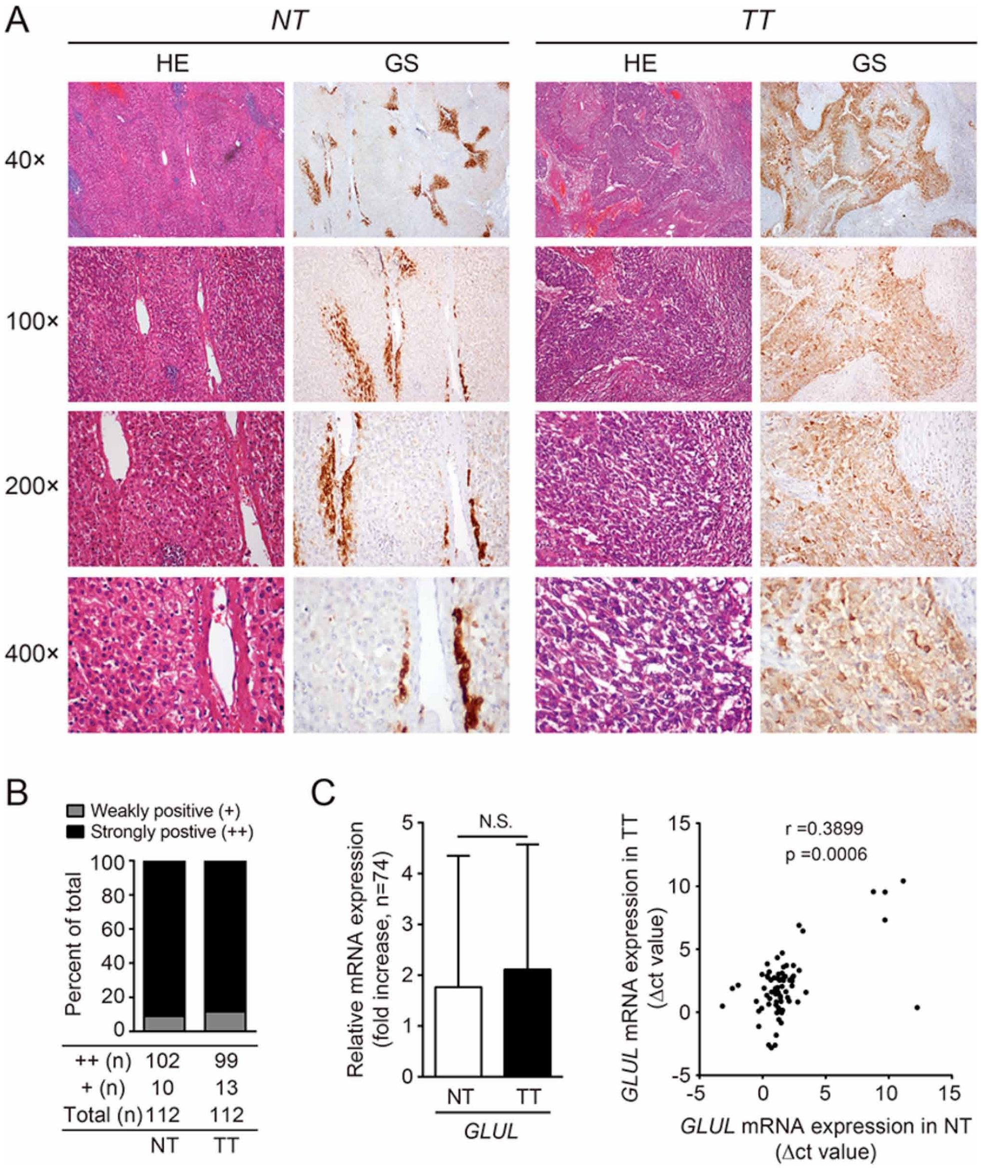

Supplementary Figure 4: Distribution and expression of GS in HCC and paired adjacent non-tumor tissue. (A) The expression intensity and distribution of GS antigen were detected by immunohistochemical staining in 112 paired HCC (TT) and their paired non-tumor tissues (NT). The representative staining of GS and their corresponding hematoxylin and eosin (HE) staining are shown. Magnifications are depicted. **(B)** The expression and intensity of total samples were evaluated and classified into three grades, ++ indicates strongly positive, + weakly positive and – negative. **(C)** The expression of *GLUL* gene, which encodes GS antigen, in HCC (TT) and paired adjacent non-tumor tissue (NT) was detected by RT-PCR (left panel, N.S. $p > 0.05$). *GLUL* mRNA expressive associations between TT and NT were evaluated using nonparametric Spearman's rank correlation analysis (right panel, $r = 0.3899$, $p = 0.0006$).

Supplementary Table 1: Clinical features of 112 HCC patients based on GLS1 and GLS2 expression

	Class	GLS1			GLS2		
		Negative	Positive	*p*	Negative	Positive	*p*
Age(years)	Mean	55.69	52.00		53.37	52.26	
	SD	11.96	9.40	0.1636	10.52	9.71	0.4431
WBC (10^9/L)	Negative	7.40	8.12		7.69	8.24	
	Positive	3.43	3.16	0.504	3.33	3.11	0.4632
Plt (10^9/L)	Negative	148.06	141.28		141.19	146.50	
	Positive	61.31	69.72	0.5041	72.44	58.71	0.5279
PT (s)	Negative	12.77	13.37		13.14	13.24	
	Positive	1.00	2.45	0.6215	2.35	1.77	0.8929
Alb (μmol/L)	Negative	35.69	35.14		35.63	34.83	
	Positive	4.53	4.28	0.602	4.81	3.52	0.589
TBil (μmol/L)	Negative	16.64	20.88		20.46	18.34	
	Positive	5.33	11.91	0.2634	10.95	9.93	0.2917
Glu (μmol/L)	Negative	5.79	5.89		5.47	6.37	
	Positive	1.81	1.73	0.8955	1.03	2.30	0.3274
AFP (ng/mL)	Negative	799.45	1100.70		780.01	1402.00	
	Positive	2504.70	2920.50	0.2563	2475.30	3273.10	0.3638
Length (cm)	Negative	5.40	5.74		5.87	5.30	
	Positive	3.33	3.29	0.5886	3.27	3.32	0.3298
Volume# (cm^3)	Negative	154.99	154.32		195.07	89.56	
	Positive	297.86	296.90	0.607	352.04	153.92	0.1568
Gender	Female	6	12		10	8	
	Male	23	71	0.557	60	34	0.5974
Diabetes	No	27	77		68	36	
	Yes	2	6	1	2	6	0.0507
Hepatitis	No	9	23		19	13	
	Yes	20	60	0.8123	51	29	0.6719
Cirrhosis	No	4	4		6	2	
	Yes	21	63	0.2061	52	32	0.7054
Necrosis	No	21	54		45	30	
	Yes	6	17	1	14	9	1
Differentiation	1	3	23		11	4	
	2	22	58		46	34	
	3	0	7	0.3054	4	3	0.5707
Tumor number	1	23	63		52	34	
	2	2	7		7	2	
	3	4	12	1	10	6	0.6875

(*continued*)

Class		GLS1			GLS2		
		Negative	Positive	*p*	Negative	Positive	*p*
Tumor capsule invaded	No	15	24		24	15	
	Yes	14	56	0.0439	46	27	1
Never invaded	No	24	69		61	32	
	Yes	0	2	1	0	2	0.1256
Micro-vessel invaded	No	18	46		40	24	
	Yes	11	35	0.6663	28	18	1
Recurrence	No	16	54		40	31	
	Yes	16	25	0.2479	27	14	0.5958
Stage	I	10	26		23	13	
	II	11	26		20	17	
	IIIA	5	21		15	11	
	IIIB	1	1		2	0	
	IVA	2	6		7	1	
	IVB	0	2	0.8205	2	0	0.3903

WBC, white blood cell; Plt, platelet; PT, prothrombin time; Alb, Albumin; TBil, Total bilirubin; Glu, Glucose; AFP, alpha fetal protein. #Tumor volume = length^2 * width/3.

Supplementary Table 2: Correlation between GLS1, GLS2, and clinical index

Index	Category	GLS1 in TT			GLS2 in TT		
		Negative	Positive	*P* value	Negative	Positive	*P* value
GLS1/2 in NT	Negative	6	24		2	3	
	Weak	9	39		4	1	
	Strong	3	9	0.021	22	42	0.0380
Gender	Female	3	10		6	6	
	Male	15	62	0.719	24	56	0.236
Grade	High	2	1		1	1	
	Medium	13	38		15	35	
	Low	3	33	0.020	14	19	0.521
Tumor number	Single	15	67		25	53	
	Multiple	3	5	0.195	5	3	0.121
T Stage	I	3	8		5	5	
	II	8	25		7	25	
	III	7	35		16	24	
	IV	0	3	0.710	1	2	0.233
Lymph node	Negative	17	67		29	51	
	Positive	0	1	1	0	1	1
Extrahepatic metastasis	No	18	69		29	54	
	Yes	0	1	1	0	1	1
Stage	1	3	8		5	5	
	2	8	25		7	25	
	3	7	36		17	24	
	4	0	2	0.706	0	2	0.138
Survival time	Mean (months)	44.56	29.32		24.37	24.73	
	SD	22.98	23.84	0.018	35.60	23.29	0.049
Age	Mean (years)	59.28	53.63		52.47	7.99	
	SD	10.42	8.03	0.014	55.86	8.86	0.084
Length	Mean (cm)	6.11	7.65		6.81	4.28	
	SD	4.16	5.35	0.259	7.68	5.59	0.466
Volume	Mean (cm^3)	92.10	134.21		91.40	132.13	
	SD	148.55	249.61	0.496	146.43	274.64	0.312

Stage the 7th AJCC.
